# Experiences and perceptions of migrant populations in South Africa on COVID-19 immunization: an interpretative phenomenological analysis

**DOI:** 10.1186/s12889-024-20562-1

**Published:** 2024-11-12

**Authors:** Ferdinand C Mukumbang, Sibusiso Ndlovu, Babatope O Adebiyi

**Affiliations:** 1 Department of Global Health, School of Public Health, University of Washington, Seattle, USA; 2 Doctors Without Borders, Johannesburg, Gauteng South Africa; 3 Centre for Interdisciplinary Studies of Children, Families and Society, University of the Western Cape, Cape Town, South Africa; 4 Section of Rheumatology, Department of Paediatrics, Cumming School of Medicine, University of Calgary, Calgary, Alberta Canada

**Keywords:** Migrant population, COVID-19, Vaccine hesitancy, Vaccine acceptance, COVID-19 vaccines, SARS-CoV-2

## Abstract

**Introduction:**

Migrant populations (asylum seekers, permit holders, refugees, and undocumented migrants) living in South Africa face various individual, social, and physical circumstances that underpin their decisions, motivation, and ability to receive the COVID-19 vaccine. We conducted a qualitative study to explore the experiences and perceptions of migrant populations in South Africa on COVID-19 vaccines to inform recommendations for improved COVID-19 immunization.

**Methods:**

We conducted an Interpretative Phenomenological Analysis (IPA) with 20 asylum seekers, permit holders, refugees, and undocumented migrants living in South Africa. We applied a maximum variation purposive sampling approach to capture all three categories of migrants in South Africa. Semi-structured interviews were conducted and recorded electronically with consent and permission from the study participants. The recordings were transcribed and analyzed thematically following the IPA using Atlas.ti version 9.

**Results:**

Four major reflective themes emanated from the data analysis. (1) While some migrants perceived being excluded from the South African national immunization program at the level of advertisement and felt discriminated against at the immunization centers, others felt included in the program at all levels. (2) Skepticism, myths, and conspiracy theories around the origin of SARS-CoV-2 and the COVID-19 vaccine are pervasive among migrant populations in South Africa. (3) There is a continuum of COVID-19 vaccine acceptance/hesitancy ranging from being vaccinated through waiting for the chance to be vaccinated to refusal. (4) Accepting the vaccine or being hesitant follows the beliefs of the participant, knowledge of the vaccine’s benefits, and lessons learned from others already vaccinated.

**Conclusion:**

COVID-19 vaccine inclusiveness, awareness, and uptake should be enhanced through migrant-aware policies and actions such as community mobilization, healthcare professional training, and mass media campaigns.

**Supplementary Information:**

The online version contains supplementary material available at 10.1186/s12889-024-20562-1.

## Background

By the end of 2022, an estimated 652 million COVID-19 cases had been confirmed, with about 6.6 million deaths globally. Socio-economically, through implemented lockdown measures, COVID-19 affected economic growth locally and globally by increasing the risks of economic instability, decreasing migration and remittance, reducing income from travel and tourism, and reducing the number of small and medium industries and informal businesses [1]. Consequently, developing a vaccine was considered the fastest and most efficient way to reduce its biological and economic threats. The failures of other COVID-19 prevention approaches such as handwashing, social distancing and quarantining highlighted the importance of COVID-19 vaccine development [2]. As such, global efforts were harnessed to produce potent, effective, and safe vaccines against the COVID-19 pandemic, which was achieved in less than a year [3].

The SAR-CoV-2 virus was first identified on December 8, 2019 [4]. By December 2, 2020, a Pfizer vaccine was first approved for emergency use. By July 2021, there were 18 vaccines approved for emergency use by at least one regulatory authority [5]. Amit et al. [6] estimated that an 85% vaccine efficacy (less infection and symptoms of COVID-19) is achieved 15 to 28 days after the first vaccine dose. A 70–85% population-level vaccination is estimated to reach “herd immunity,” with booster shots needed to keep the virus in check [7]. Individuals vaccinated who experience COVID-19 symptoms are 44% less likely to be hospitalized, and 51% are less likely to die than unvaccinated individuals [8].

As of December 14, 2021, nearly one billion individuals globally were partially vaccinated, and another 3.64 billion were fully immunized against COVID-19. However, over 44% of the world, predominantly in low- and middle-income countries (LMIC), were still unvaccinated [8]. In September 2022, only 22% of the population in Africa received two COVID-19 vaccine doses [9]. In December 2022, an estimated 39.9% of people living in South Africa had taken one dose of a COVID-19 vaccine, and only 35.0% had been fully vaccinated, representing half the WHO target of 70% to achieve herd immunity.

The WHO proposed an equitable distribution framework to achieve maximum benefits following the development of COVID-19 vaccines in 2020. According to WHO’s COVID-19 vaccine allocation mechanisms, access to the vaccines at its inception would protect healthcare workers and the most at risk from the pandemic’s public health and economic impacts [10]. The goal changed to vaccinating 70–85% of the world’s population to achieve herd immunity [11]. When vaccines became available, many high-income countries first secured them for their populations and then redistributed them to the most vulnerable everywhere [10]. Most LMICs depended on the COVID-19 Vaccines Global Access (COVAX) Facility to obtain vaccines, which failed to meet its goals consistently [12]. Consequently, inequalities in COVID-19 vaccine distribution arose among different countries and sub-populations [13].

The International Rescue Committee reported in May 2021 that 60% of countries receiving their vaccines through COVAX had excluded some migrant populations from their national vaccination plans [14]. Several studies have highlighted the challenges of migrant groups—asylum-seekers, refugees, and undocumented migrants—during the COVID-19 era [15], warranting their consideration as an at-risk population for the COVID-19 vaccine prioritization [10]. The COVID-19 era exacerbated the migrant population’s vulnerabilities: weakened social support structures, socioeconomic difficulties, unequal access to healthcare and social services, precarious living and working conditions, and higher risks of exploitation and abuse [10, 16]. These enhanced vulnerabilities have led to the systemic marginalization of these migrant populations [17], raising concerns about their equitable access to COVID-19 vaccination [18].

With the constant mutating of the SARS-CoV-2 virus and the emergence of new variants, achieving high vaccination rates across all populations becomes critical [19]. Nevertheless, vaccine hesitancy—delay in accepting or refusing vaccines despite the availability of vaccination services—remains a considerable concern as it hampers the efforts to achieve herd immunity. A recent global study showed a 24.8% COVID-19 vaccine hesitancy in 2021, associated with a lack of trust in COVID-19 vaccine safety and science and skepticism about its efficacy [20]. Studies have also shown a disparity in COVID-19 vaccine uptake between migrant and non-migrant populations, with migrant populations having low uptakes of up to 8% in High Income Countries [14, 21]. While migrants might have perceived access to COVID-19 vaccines, their demand or uptake remains suboptimal [18].

The immunization rates for the overall population in top refugee-hosting countries range from 77% in Germany to just 13% in Sudan. The WHO released a new operational guide to promote COVID-19 vaccination uptake and tackle vaccine hesitancy (by addressing access to vaccines) among migrant populations, proposing the inclusion of migrant populations in the vaccine prioritization [22]. In the context of barriers to accessing vaccination, especially in LMIC, and attitudes towards COVID-19 immunization [23], we aimed to explore the experiences and perceptions of migrant populations in South Africa on COVID-19 immunization.

While there is a general attitude of vaccine hesitancy in South Africa, especially among those living in the townships, Steenberg and colleagues emphasized the need for more social and behavioral research in different population groups in South Africa to understand culture-specific issues around COVID-19 vaccine uptake and hesitancy [24]. Improving our understanding of the dynamics around COVID-19 acceptance/hesitance among migrant populations can help design strategies to address the low vaccination rates among these populations. Our knowledge can also help strengthen the health systems of LMICs for future pandemics of a similar nature.

## Methodology

We conducted an Interpretative Phenomenological Analysis (IPA). IPA is a qualitative research approach that explores how participants make sense of their personal and social worlds [25, 26]. An IPA study focuses on unearthing how the participants make meanings of experiences and events (or non-events). IPA is underpinned by the philosophies of phenomenology, hermeneutics, and idiographic stance [27]. Phenomenologically, it focuses on lived experiences and is concerned with an individual’s perception, appraisal, or account of a situation or an event [25]. Hermeneutically, IPA embraces the participants’ notion of interpretation and sensemaking because humans are sense-making beings [25]. In this way, the researcher tries to make sense of the participants and what is happening to them [25]. IPA’s idiographic properties warrant that the researcher examines the detailed experience of each case before moving to more general claims [25].

We adopted IPA to focus on the experiences and perceptions of migrants in South Africa, how they translate to how they are treated, and their interpretation of situations regarding taking the COVID-19 vaccine. All three authors of the work are migrants from Cameroon, Nigeria, and Zimbabwe, and they have experiences and, consequently, formed interpretations and perspectives on the phenomenon. To this end, while conducting this study, we applied the tenets of conducting a phenomenological study, epoché or bracketing—eliminating preconceptions that may taint the research process [28]. The methodological consequences of adopting principles from descriptive phenomenology are that we could allow participants’ experiences and perspectives to appear naturally [28] and reflect these experiences to conceptualize the notion of COVID-19 immunization in migrant communities [29].

## Methods

### Study setting

With an estimated net immigration of 1,02 million people between 2016 and 2021, South Africa has the highest rates of cross-border migration, estimated at 2.9 million migrants [30]. The Gauteng and Western Cape provinces of South Africa received the highest number of in-migrants between 2016 and 2021, respectively, as they offer better economic, social-political, cultural, or environmental conditions than the other seven provinces [30]. We focused on these two provinces while selecting participants for the study.

The COVID-19 containment measures adopted by the SA government through the lockdown of the nation tremendously deepened the unequal treatment of asylum-seekers and refugees in South Africa. This inequity can be seen through the South African government’s lack of consideration of this marginalized population in economic, poverty, and hunger alleviation schemes during the national lockdowns imposed by the government to curb the spread of the COVID-19 pandemic [31]. Despite this position, other countries worldwide have committed to include migrant populations in their immunization program. Nevertheless, efforts to honor this commitment in South Africa are hampered by a seriously compromised Home Affairs Department (exacerbated by the 2020–2021 lockdown), making many migrants in South Africa struggle to regularize their stay.

Individuals must be registered on the Electronic Vaccination Data System to be vaccinated in South Africa. The system requires each person to have an identification number so the government can track who is vaccinated, record which vaccine each person received, and follow up with an individual if needed. Vaccination centers require an identity number, a passport number, or a refugee permit to register, leaving no option for those without these documents. Furthermore, no clear directive exists on how undocumented migrants can register for vaccination. Consequently, access to COVID-19 vaccines for migrant populations is limited. Fear of being repatriated based on unregularized status also constitutes a huge barrier to using the facilities offered for immunization [32].

### Study design

We conducted an IPA to understand the experiences and perceptions of South African migrant populations on the uptake of COVID-19 vaccines. IPA is a suitable approach for discovering how individuals perceive their situations and make sense of their personal and social worlds [26].

### Population, sampling, and data collection

Purposive and snowball sampling was used to recruit study participants (asylum seekers, permit holders, refugees, and undocumented migrants). We purposefully recruited a diverse group of 20 participants based on family structure, gender, age, socioeconomic status, culture, and employment status. To be included in the study, the participant had to be.

- Between the ages of 18 and 65.
- A migrant from a sub-Saharan country except for South Africa,
- An asylum-seeking or refugee status.
- They can express themselves in French or English; a translator is available if they cannot speak.

We only considered including participants between the ages of 18 and 65 to avoid issues of consenting and potential exploitation, as migrants are already considered a vulnerable population. Including minors having immigrant status increases their vulnerability [33] consequently, they were excluded from this study. Our focus on migrants from sub-Saharan countries was based on the reports that they are the most alienated population in South Africa, facing the brunt of xenophobic attacks and unfair treatment from the South African government [34, 35]. To this end, we excluded migrants from other African countries and those of other continents as they face comparatively lesser xenophobic alienation than their sub-Saharan counterparts [36]. Being of asylum-seeking or refugee status was critical for their inclusion, as the study focuses on their experience with COVID-19 vaccination. To capture varied perspectives and experiences of migrants in South Africa, we included Francophonie African migrants. There is evidence suggesting that francophone and other ‘continental’ African migrants face issues of language barriers and interactions in South Africa [37, 38].

The Tshwane Migrant Project started on July 12, 2019, to provide a supportive, specialized HUB for undocumented migrants, asylum seekers, permit holders, and refugees living in Tshwane. Participants were recruited via two avenues: direct contact and snowballing. For direct contact, SN, who worked as the Health Promotion Supervisor at Médecins Sans Frontier, Tshwane Migrant Project, recruited participants through the project. After submitting the research proposal and obtaining ethics clearance from the University of the Western Cape ethics board, we received permission from the Tshwane Migrant Project to conduct the research. SN presented the study aims and objectives to the migrants living at the HUB at the Sediba Hope Medical Centre in Tshwane CBD. The HUB offers free and confidential primary health care services, mental health care, and referrals for secondary or specialized care to people who are often unable to access appropriate health care or social services. Twelve participants volunteered to take part in the study through the project. Table 1 provides information about the participants.

**Table 1:** Participant characteristics

Characteristics	Participants (*N* = 20)
**Gender**	
Male	12
Female	8
**Country of Origin**	
Tanzania	1
Malawi	3
Rwanda	2
Congo DRC	5
Zimbabwe	6
Cameroun	1
Somalia	1
Nigeria	1
**Immigration status**	
Asylum seeker	11
Undocumented	5
Permit holder	3
Refugee	1
**Qualification**	
Masters	3
Degree	5
High school	9
Primary school	3
**Age**	
20–30	6
31–40	5
41–50	9
**Occupation**	
Student	5
Shop assistant	1
Unemployed	7
Technician	1
Trader	5
Community health worker	1

As Nwoke et al. [32] noted, migrants living aboard already have established social networks in the community (immigrant organizations, cultural associations, and religious institutions). For the snowballing recruitment, FCM and BOA contacted the leaders of two known migrant social gatherings living in Cape Town for permission to present the aim and objectives of the study to their group members. The first seed participants, obtained through those mentioned above from the social groups, were asked to invite other migrants who fit the study criteria.

When in contact with a potential participant, we undertook the following process. (1) Explained the study’s aim and objectives and the roles of the potential study participant through a phone call. (2) Participants who were willing to participate confirmed their immigration status (asylum seekers, permit holders, refugees, and undocumented migrants). (3) Participants whose immigration status was confirmed were requested to sign the study consent form and email it to the research team.

### Data collection

The goal of data collection in IPA is to enable the participant to recount as complete an account as possible of their experience and perceptions. Smith and Osborn [26] recommend using semi-structured interviews for data collection in an IPA study. Nevertheless, social distancing regulations constrained our ability to conduct only face-to-face in-depth interviews. To this end, we completed the face-to-face semi-structured interviews while observing all the social distancing protocols or via WhatsApp. For in-person interviews, we allowed the study participants to decide where the interviews should take place—University study and social spaces, workplace offices, business centers, and the HUB as needed.

We used a piloted interview guide to ensure consistency across the respondents and remain within the research aim’s ambit. The questions included (1) the participants’ opinions on COVID-19 immunization. (2) Their knowledge and perceptions of the general conspiracy theories and myths in their communities. (3) Their willingness to take the COVID-19 vaccine. (4) Their perception of whether migrants are being considered part of the COVID-19 immunization program in South Africa. (5) Possible reasons for their willingness or hesitancy to take the COVID-19 vaccine. The study’s interview guide is labeled as Supplementary file 1.

NS (MPH) conducted the interviews in the Gauteng Province, while FCM (PhD) and BOA (PhD) conducted the interviews in the Western Cape Province. The interviews were either in French or English, depending on which one the participant preferred. Each interview lasted between 30 and 45 min per participant or family unit. Permission was sought from each participant to record each interview session, and a transcription specialist transcribed each session verbatim. BOA checked all the transcriptions for appropriateness and made the necessary corrections or adjustments. The two interviews in French were also translated into English by the transcription specialist, who was fluent in English and French. All transcripts were de-identified using pseudonyms and prepared for analysis. The transcripts were uploaded onto Atlas.ti version 9 for analysis.

### Data analysis

Data analysis in IPA requires that the investigator engage in an interpretative relationship with the transcripts. To this end, we applied a form of thematic analysis based on the subjective lived experiences of the individuals [27]. We conducted IPA in a six-stage process. (1) Familiarize with the data by reading and re-reading the transcribed data. (2) Identifying the researcher’s orientation and potential bias—phenomenological reduction. (3) Identifying significant experiences and relationships of the migrants about COVID-19 vaccine uptake (4). Identifying emerging themes entails considering the micro-level data alongside the macro-level interpretation (5). Clustering themes and identifying emerging superordinate themes [27].

FCM and BOA conducted the data analysis. They each read the individual transcripts to familiarize themselves with the participants’ different narratives. Then, they met to discuss the narratives while identifying significant experiences and perceptions of the migrants about COVID-19 vaccine uptake. They also discussed and documented their different biases. Both authors developed a codebook based on a preliminary analysis of the transcripts containing the significant experiences. BOA used the code book to code the rest of the transcripts. Discursive meetings were held with FCM to identify emerging themes, which entails considering the micro-level data alongside the macro-level interpretations. They worked on clustering the emerging themes to identify superordinate themes.

## Rigor and trustworthiness

A piloted interview guide was used to conduct the study. The guide was piloted using five participants, during which time the guide was revised to ensure that the questions asked were clear enough to elicit the desired information. All three authors conducted the interviews. They all have extensive experience in conducting qualitative interviews with vulnerable populations.

Rigor is ensured through an in-depth description of the sampling, data collection, and analysis processes. Further, rigor was provided by having two investigators involved in the data analysis. The investigators engaged in a discursive process to reconcile coding differences and reflect on the developing themes. Coding was done independently using some transcripts through an iterative approach to developing a codebook. The codebook was revised further through the coding of additional transcripts.

Trustworthiness in phenomenological studies also requires ‘bracketing’ the researchers’ preconceptions as they might be essential to the data collection and interpretation processes [27]. Avoiding the ‘voices’ of subjectivity from interfering with the authenticity of what the research respondents essentially mean in their accounts requires the application of phenomenological reduction [28]. Phenomenological reduction requires stepwise implementation of bracketing. While applying bracketing, BOA and FCM continuously managed intrusions of their preunderstandings throughout the research, as advised by Finlay [26].

## Results

**Fig. 1:**
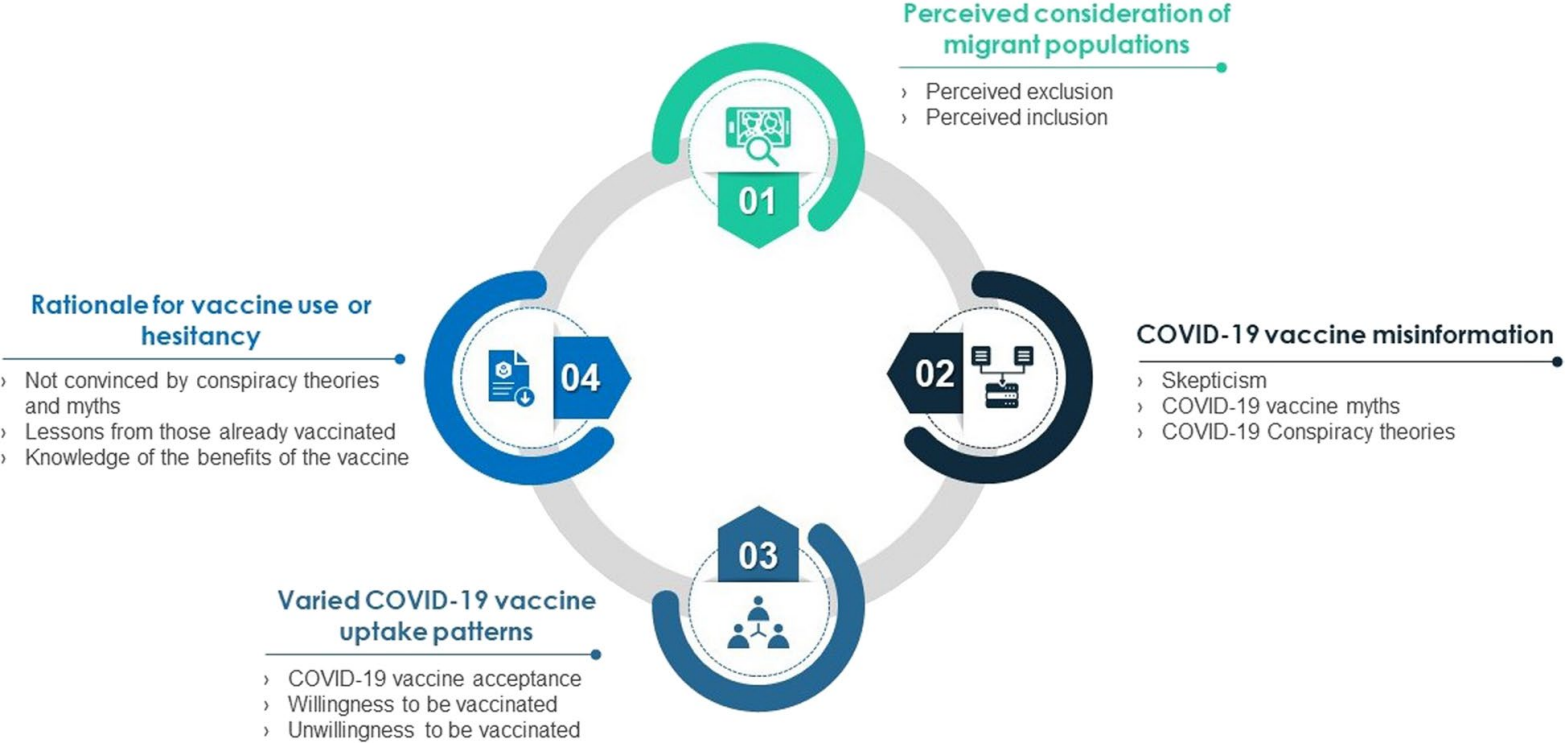
Thematic representation of migrants’ experiences and perceptions on COVID-19 immunization

 The experiences and perceptions of the migrant populations in South Africa on COVID-19 immunization were captured in three main themes: (1) Perceived consideration of migrant populations in the national COVID-19 vaccination program, (2) COVID-19 vaccine misinformation fueling skepticism, myths, and conspiracy theories, (3) variability in COVID-19 vaccine uptake and (4) Rationale for vaccine use or hesitancy. Figure 1 is an illustration of the thematic representation of the experiences and perceptions of the migrant populations on COVID-19 immunization.

### Perceived consideration of migrant populations

The experiences and perceptions of migrant inclusion/exclusion in the South African COVID-19 immunization program were identified at three levels: the COVID-19 vaccination centers, the advertising, and the presidential levels.

#### Perceived exclusion

The study participants believed that migrants were being excluded from accessing COVID-19 vaccines. One participant perceived discrimination at one of the COVID-19 vaccination centers:

But some nurses said you guys want to get all our vaccines. Go back to your countries and get vaccinated… You can see that segregation, saying, ‘No, you’re not a South African.’ So, I was uncomfortable going for the second jab because of those statements [Voice 15; Permit Holder; Zimbabwe].

Participants also perceived exclusion from the messaging by the South African government and the media. A participant suggested that the information suggesting the inclusion of migrants in the immunization program is minimal.

No. Because I’m concerned about the smaller information that is circulating towards migrants. Why? Because I haven’t seen an advert on television considering the migrants [Voice 3; Zimbabwe; Undocumented].

One other participant further explained that how the President addresses the public about the COVID-19 immunization program suggests that migrant populations are excluded.

When the President addresses people, he will say my fellow South Africans. So, maybe the thing shows he is not including us [Voice 7; Zimbabwe; Undocumented].

#### Perceived inclusion

While others felt that they, as migrants, were being excluded from the national COVID-19 immunization program at various levels, others conceded that migrants were not being excluded based on their experiences and observations.

It was a very good decision to let asylum seekers get vaccinated because it’s about saving lives. [Voice 10; Rwanda; Asylum Seeker]

No, no, no, as a foreigner, there are no barriers to vaccination; it is open to everyone. Because that’s the vaccination [Voice 16; Congo DRC; Refugee].

Another participant suggested that the government is doing more to promote the uptake of COVID-19, even among migrant communities.

The government they are doing more. They promote health on the street. I saw many, many CHW [Community Health Workers]. Also, from the health department. So, as we promote health, we tell them [migrants] to go to the nearest clinic [Voice 12; Malawi; Asylum Seeker].

Another participant even suggested that migrants were incentivized to go for the COVID-19 vaccines by offering them food parcels after vaccination.

If you want to vaccinate, they give them food. Guys, if you want to vaccinate, there’s also food after vaccination, and with the parcel of food, go [Voice 16; Congo DRC; Refugee].

### COVID-19 vaccine misinformation

COVID-19 misinformation affects the migrant population in South Africa, manifesting in the forms of (1) skepticism based on vaccine side effects and safety, (2) COVID-19 vaccine myths, and (3) conspiracy theories.

#### Skepticism

Skepticism around the COVID-19 vaccine emanates from false claims about the adverse side effects of the COVID-19 vaccine. One participant indicated their skepticism in the following:

First, you will see Astra Zeneca. You will see a lot of complaints in a lot of countries. This is what from Astra has reinforced my skepticism because I was very skeptical [Voice 13; Congo DRC; Asylum Seeker].

Other participants suggested that the COVID-19 vaccine skepticism emanated from the speed with which the vaccine was manufactured and approved for emergency use.

They [migrants] don’t believe in that vaccine. I don’t believe research has been conducted to test it. Isn’t it too early? [Voice 1; Tanzania; Permit Holder].

The challenge is that people don’t want to go and get the vaccine because they say it’s [the vaccine] not approved [Voice 6; Zimbabwe; Undocumented].

#### COVID-19 vaccine myths

Besides the skepticism based on side effects and the speed with which they were developed, there are also myths about the impact of the COVID-19 vaccine.

A prevalent myth is that the vaccine kills those who receive it after different time frames.

Others say that if you get a vaccine for just two years or some months, you want to die [Voice 6; Zimbabwe; Undocumented].

Some say you’ll die if you take this vaccination [Voice 9; Congo DRC; Asylum Seeker].

They are testing people, African people, to die after five years [Voice 12; Malawi; Asylum Seeker].

Other myths were less extremist, not leading to death but affecting their sexual performance and sometimes causing sterility among those who have taken the vaccine.

Some they are saying for us meaning, you know if you are injected, sexually you are destroyed.

I’m not going to get in babies again. I’m not reproducing babies [Voice 12; Malawi; Asylum Seeker].

On the men’s side, they are saying when you get the vaccine, the men cannot function; loss of sexually as they used to function [Voice 10; Rwanda; Asylum Seeker].

Other participants suggested that the COVID-19 vaccine is a tracking device that is being introduced into humans.

When you have injected something cheap that is introduced into your body, they will know where you’re going [Voice 3; Zimbabwe; Asylum Seeker].

Some myths relate to the vaccine ‘changing’ the person. One of the participants recounted that they had heard that the vaccine causes hypertrichosis – body hair growth.

When you get the vaccine, it will change you… the people they’ll grow hair everywhere on the body [Voice 3; Zimbabwe; Asylum Seeker].

#### COVID-19 conspiracy theories

The COVID-19 conspiracy theories relate to the origin of COVID-19, the development agenda, and the reason behind mass immunization. As with the myths, several conspiracy theories are also trending among the migrant populations in South Africa.

Regarding the origin of COVID-19, some participants suggest that the USA created it to destabilize the Chinese economy.

The Americans wanted to reduce the rise of China, so they had to reduce their economic growth, so they injected this virus to destabilize China [Voice 13; Congo DRC; Asylum Seeker].

Others suggest that installing 5G network antennae worldwide is responsible for the COVID-19 pandemic.

Another version was also the G5. The G5 antennas that they are installing are the antennas that give the [COVID-19] disease [Voice 13; Congo DRC; Asylum Seeker].

I don’t know if some of the G4 and G5 networks are Wi-Fi networks [Voice 16; Congo DRC; Refugee].

Another conspiracy theory surrounding the COVID-19 pandemic relates to the pandemic being intentionally orchestrated by Bill Gates for various reasons.

There is still another version… we say that Bill Gates wants to inject this to sell the vaccine that it was for financial reasons [Voice 4; Zimbabwe; Undocumented].

Bill Gates was talking about decreasing the population could be [COVID-19 pandemic] also related to this one [Voice 16; Congo DRC; Refugee].

Others have suggested that the COVID-19 pandemic and the vaccine aim to reduce the African population.

To decrease the African population because the number is increasing a lot and the European population is already old. They are not fertile [Voice 13; Congo DRC; Asylum Seeker].

I still have that mentality that the West is afraid of the population of Africa, and the only sane way to depopulate Africa is through diseases [Voice 17; Permit Holder; Nigeria].

There are also versions related to mistakes that occurred in the laboratory, which caused the current COVID-19 pandemic.

I still think it was a mistake in the lab because there’ve been variants of COVID [Voice 17; Permit Holder; Nigeria].

There was a leak in the laboratory. They were doing the tests, and by recklessness, the bacteria [SIC] escaped [Voice 13; Congo DRC; Asylum Seeker].

### Varied COVID-19 vaccine uptake patterns

Some participants reported that they had already received the COVID-19 vaccine, while others were still contemplating it. Nevertheless, others were utterly unwilling to be vaccinated.

#### COVID-19 vaccine acceptance

Some participants indicated that they had already been vaccinated during the interviews.

Yes, I consider it because I’m already vaccinated [Voice 7; Zimbabwe; Undocumented].

I did get vaccinated a few months ago [Voice 10; Rwanda; Asylum Seeker].

#### Willingness to be vaccinated

Some showed a willingness to take the vaccine once it was available or they could take it.

I wanted earlier, but they haven’t called me, or they haven’t sent me the message [Voice 7; Zimbabwe; Undocumented].

Yeah, given a chance, I will do [Voice 3; Zimbabwe; Asylum Seeker].

Others suggested that they preferred to wait longer before taking the COVID-19 vaccine.

For me, I prefer to wait until next year, when I can get an injection next year [Voice 7; Zimbabwe; Undocumented].

I’m still deciding how I can take it. Because as the way it goes it [Voice 4; Zimbabwe; Undocumented].

#### Unwillingness to be vaccinated

Some were unwilling to consider taking the vaccine when the interviews were conducted.

I didn’t even register, but I haven’t. Because they said you register first, they’ll send you the message. I don’t want to [Voice 14; Congo DRC; Asylum Seeker].

Really? No, I’m not really convinced that I should vaccinate myself. Why? Because I don’t see it being of value even if I get vaccinated [Voice 17; Nigeria; Permit Holder].

### Rationale for vaccine use or hesitancy

The participants offered different reasons for accepting the vaccines or being hesitant. These included (1) unbelief in conspiracy theories and myths, (2) Knowledge of the benefits of the vaccine, and (3) Lessons from others already vaccinated.

#### Not convinced by conspiracy theories and myths

Most of the participants who received the vaccine expressed their disbelief in conspiracy theories.

I don’t think that [myths] are true. Because all the scientists have said that the vaccine doesn’t cause all those things, it’s a people’s story [Voice 10; Rwanda; Asylum Seeker].

One participant said he did not believe in conspiracy theories, especially those relating the COVID-19 pandemic to the anti-Christ and the end of days.

I heard people say that God struck people because of sins or that it is anti-Christ, but I did not believe in it [Voice 13; Congo DRC; Asylum Seeker].

#### Lessons from those already vaccinated

Other participants suggested taking the vaccine because they observed that others had taken the COVID-19 vaccine and were not negatively affected.

I think the vaccine came a little later, after so many months of COVID-19. By the time we had the vaccine, I think I was ready for it. But if it were administered, maybe the first two or so months, I would not take it [Voice 20; Congo DRC; Asylum Seeker].

Some participants even suggested that it would be encouraging for government officials to openly take the vaccines as an example for others to follow.

The big people in the government should do it [get vaccinated] openly and say you should vaccinate so that when, for example, I see like the Minister is doing it [getting vaccinated] on air, it will dispel the myths [Voice 18; Cameroon; Asylum seeker].

#### Knowledge of the benefits of the vaccine

Some participants reported that they understood the necessity of the vaccine and what the vaccine was designed to achieve.

One benefit is that I’m going to protect myself, and I’m going to be able to walk anywhere [Voice 16; Congo DRC; Refugee].

I say this [immunizing] will only increase your immunity, which will be very important to take it [Voice 19; Zimbabwe; Undocumented].

Other participants knew the vaccine does not prevent one from contracting COVID-19 but can reduce its symptoms and chances of hospitalization.

If you vaccinate, it doesn’t mean you won’t get the Corona, but it won’t have power in your body. To give your protection [Voice 17; Nigeria; Permit Holder].

It will help many people who took the vaccination or were not hospitalized [Voice 12; Malawi; Asylum Seeker].

Other participants understood that the COVID-19 vaccines serve as a protection for themselves and others.

And I always want to emphasize it with those who do it for your protection. You do it yourself to protect others [Voice 10; Rwanda; Asylum Seeker].

Some participants understood that the COVID-19 vaccine also reduces the potential spread of the COVID-19 pandemic.

I don’t want to spread the disease. And also, I don’t want to get infected again [Voice 2; Malawi; Asylum Seeker].

To reduce the spreading and affecting people more [Voice 12; Malawi; Asylum Seeker].

## Discussions

We conducted a qualitative study to explore the experiences and perceptions of migrant populations in South Africa on COVID-19 vaccines to inform recommendations for improved COVID-19 immunization. Four major reflective themes were identified. (1) While some migrants perceived being excluded from the South African national immunization program at the level of information shared on the immunization program and felt discriminated against at the immunization centers, others did not perceive being excluded from the immunization program. (2) Through misinformation, there is a rife of skepticism, myths, and conspiracy theories around the origin of SARS-CoV-2 and the COVID-19 vaccine among migrant populations in South Africa. (3) There is a continuum of COVID-19 vaccine acceptance/hesitancy ranging from being vaccinated through waiting for the chance to be vaccinated to refusing. (4) Vaccine acceptance/hesitancy relates to the participant’s beliefs, knowledge of the vaccine’s benefits, and lessons learned from vaccinated individuals.

Regarding the perceived consideration of migrant populations for the COVID-19 immunization program in South Africa, there was a mixed perception of whether the migrants were included or excluded from the COVID-19 vaccination program in South Africa. The exclusion was reported at the information-sharing platforms and immunization centers. Mukumbang [15] also noted that in South Africa, despite having a large population of French-speaking migrants within their borders, COVID-19-related adverts were never translated or subtitled in French, constituting an aspect of non-inclusion. Lack of accessible information in an appropriate language was also reported in a qualitative study conducted among migrants living in the UK [39]. A survey conducted in June 2021 by 52 national Red Cross and Red Crescent societies found that 90% of migrants reported a lack of information or awareness on where and how to access COVID-19 vaccines, and 67% identified language barriers [22]. To improve the inclusion and participation of the migrant population in the COVID-19 vaccination campaigns, the South African government should consider using inclusive language in framing the communications the COVID-19 immunization. Languages such as French and Shona, spoken by larger migrant populations, should also be included in the formal South African languages. Anti-discriminatory and migrant-aware training programs should also be provided to healthcare providers at immunization centers to enhance their sense of inclusivity.

Regarding the willingness to take the COVID-19 vaccine, we found varying sentiments on COVID-19 acceptability among the migrant populations. COVID-19 acceptance and hesitancy variation have also been reported among migrant populations [40, 41]. Vaccine hesitancy, which involves varying levels of doubt, indecision, uncertainty, or mistrust about vaccination [42], accounts for the variability in the willingness to immunize. Although moderate COVID-19 vaccine acceptance levels have also been reported among migrant populations [43], (mis)trust in host governments has also been frequently cited as a concern, especially by undocumented migrants [44]. We also found that some migrants were willing to take the vaccine but were waiting for the opportunity. Page et al.. found a mismatch between perceived acceptability and uptake of the COVID-19 vaccination in migrant populations [18]. Crawshaw et al. [45] suggest that the discordance between perceived vaccine acceptability and vaccine uptake can be addressed by considering the different migrant populations within the existing vaccine priority structure defined by individual countries and tailoring targeted approaches based on their specific risk factors for under-immunization. For instance, addressing language barriers among francophone migrants through outreach initiatives can contribute to effective information sharing and address mistrust of the local authority.

Our study unveiled that skepticism based on the side effects of the COVID-19 vaccine, myths about the COVID-19 vaccine, and conspiracy theories about the origin of the COVID-19 pandemic are rife among the migrant populations of South Africa. It has been argued that South Africa presents context-specific conditions that amplify uncertainty related to the nature of communicability, which enforces racialization and thus exacerbates existing societal polarizations [46]. Migrant communities are reported to be more susceptible to COVID-19 vaccine misinformation, mainly where language barriers and social exclusion contribute to a deficit of accurate information [45]. Lack of information from a trusted source and inappropriate language has also been identified as significant determinants of COVID-19 vaccine acceptance/hesitancy [40]. Ullah and colleagues noted that anti-COVID-19 vaccine controversies about vaccine safety are rapidly circulating on social media via different platforms, especially in the few months after the vaccines were approved for emergency use [47]. In fact, during the infodemic—information overload—phase of the COVID-19 pandemic, there was also an upsurge of conspiracy theories about the virus’s origins and suspicions around the motives behind government COVID-19 control measures among different communities globally [48]. Pertwee et al. suggested that conspiracy theories and myths about COVID-19 and vaccines are not merely false beliefs but an expression of widespread fears and anxieties emerging in acute social uncertainty [48].

Similar to Steenberg et al.‘s [46] observation that Africanized forms of conspiracy theories were underpinned by colonization and racism, our study confirms that skepticism among the migrant population about the COVID-19 pandemic and the vaccine was also related to their immigration status and xenophobia. Skeptical attitudes towards vaccination have also been used to explain the vaccine hesitancy end of the COVID-19 vaccine acceptance/hesitancy continuum observed among migrant populations [22]. Indeed, Enders et al. found that COVID-19 vaccine skepticism, myths, and conspiracy theories have detrimental effects on the uptake of the current COVID-19 vaccines and their boosters—vaccine hesitancy and refusal [49]. To address the challenges of skepticism, myths, and conspiracy theories, the South African government should consider implementing accessible information campaigns in major migrant population languages, such as French, on COVID-19 vaccine side effects and contents. Such information campaigns should also counter misinformation addressing myths and conspiracy theories delivered through trusted community sources such as NGOs, migrant community groups, and religious groups [39]. Such information campaigns must be sensitive and culturally appropriate and risk stigmatizing individual communities, which could enforce their mistrust and disengagement [45].

We found that migrants living in South Africa develop their vaccine beliefs and formulate their COVID-19 perspectives, which impacts their vaccine acceptance/hesitancy through their life experiences and culture, structural conditions, personal background, religion, and politics. Crawshaw et al. [45] reported that these beliefs, perspectives, and attitudes could stem from longstanding structural inequities, stigma, discrimination, exclusion, and lack of access to health information [22]. These structural barriers can exacerbate distrust in government while creating alienation from public health services [22]. Discriminatory incidences and the unfair application of the Siracusa principles experienced by the migrant population during the COVID-19 lockdown and restitutive actions taken by the host governments led migrants to adopt attitudes of resilience [15, 50]. These structural conditions shape migrants’ reactions to the information they receive about COVID-19 and its vaccine. According to Cooper et al. [42], vaccine myths and conspiracy theories are often rooted in distrust of institutions and associated historical and contemporary experiences of inequality, injustice, and exploitation. Changing the perspectives of migrant populations can be achieved by strengthening the collaborations with local government, the different migrant community groups, civil society groups such as Scalabrini, public health teams, and healthcare professionals to develop engagement strategies. Actively involving migrant communities in planning and implementing the COVID-19 strategy can enhance trust and encourage widespread participation in COVID-19 vaccination programs [39].

Considering the structural socioeconomic, historical, and cultural elements informing people’s vaccination choices, Storer et al. [42] proposed a shift in emphasis toward equitable principles of engagement. Paradoxically, Storer et al. [42] found that despite restrictive measures, many within migrant groups initially described as ‘vaccine-hesitant’ changed their minds and became vaccinated. They discovered that vaccine acceptance was not attributed to successful civic engagement. Instead, it indicated deep distrust in science and the host government—compliance with vaccine mandates to continue their social and economic activities [42]. Such distrust can explain vaccine hesitancy among some of the study participants. Therefore, as Cooper [36] argued, it is critical to consider the social worlds of migrants and place them at the center of efforts to reduce hesitancy and promote COVID-19 vaccine acceptance.

## Conclusion

Asylum seekers, permit holders, refugees, and undocumented migrants) Living in South Africa, people face various individual, social, and physical barriers that underpin their decisions, motivation, and ability to receive the COVID-19 vaccine. Migrant populations perceived being excluded from the South African immunization program despite efforts to include them. Skepticism, myths, and conspiracy theories around the origin of SARS-CoV-2 and the COVID-19 vaccine are rife in-migrant communities in South Africa. The feeling of marginalization and exclusion from COVID-19 relief efforts during the lockdown period in South Africa engendered mistrust in the South African government, constituting a source of COVID-19 vaccine hesitancy. Community mobilization efforts, health care professional training, non-monetary incentives, and media campaigns to enhance knowledge and awareness about vaccinations and immunization.

## Supplementary Information

Supplementary Material 1.

Supplementary Material 2.

## Data Availability

All data generated or analysed during this study are included in this published article.
