# Supplementary material for: Experiences and perceptions of migrant populations in South Africa on COVID-19 immunization: an interpretative phenomenological analysis
Source: BMC Public Health. 2024 Nov 12;24:3126. doi: 10.1186/s12889-024-20562-1 (PMC11555971; doi:10.1186/s12889-024-20562-1)
Supplement: Supplementary file 1 — Supplementary Material 1. [file 12889_2024_20562_MOESM1_ESM.docx]

**Experiences and perceptions of migrant populations in South Africa on**

**COVID-19 Immunization: An Interpretative Phenomenological Analysis**

**INTERVIEW GUIDE**

**Access to food-related questions**

- Describe how you manage to feed yourself and provide food for your family during the lockdown?
- Compare your eating pattern before and during the lockdown

Probe: How many times did you people manage to eat in a day?

- Describe the assistance that you received (food packages, vouchers etc) from any of the charity organizations or the government during the lockdown?
- Describe how NGO and government include asylum and refugee in their lockdown relief programmes
- What kind of help or support did you receive from any other person such as families, friends and neighbors? Probe: How often did you receive this help?
- Describe the kind of work you do before and during lockdown?
- Describe the type income you were receiving during the lockdown? Probe: Were you employed before and during the lockdown and at the moment?
- Describe the kind of savings (cash and kind) you have the lockdown (savings in your bank account before the lockdown)
- What made things difficult for you during the lockdown?
- Describe what would have make things easy for you doing the lockdown?

**Access to healthcare-related questions**

- Can you describe the symptoms of the Corona virus? Probe: Have you or anyone you know had any of those symptoms?
- Describe what you need to do and where to go if you have the symptoms?
- Describe how easy or difficult is it for you or anyone know to get tested for the Corona virus?

**Vaccine related questions**

What is your opinion about the COVID-19 vaccine (What do you think about the COVID-19 vaccine?

What are some of the theories (stories) you have heard about COVID-19 and the vaccine and the controlling of the world and African population?

Do you believe in some of these theories (stories) and why (or why not)?

Do you think these stories have evidence and what kind of proof have you come across?

Does such information have an impact on your beliefs around the COVID-19 pandemic?

How does such information affect your willingness to take the COVID-19 vaccine?

According to the South African government, anybody irrespective of their immigration status can take the COVID-19 vaccine when their age range is announced. Would you be willing to take the vaccine?

Do you plan to take the vaccine? If not, why would you not want to take the vaccine?

What are some of the benefits in your opinion of taking the vaccine?

There are many messages going on through various social media that the COVID-19 vaccine has magnetic properties and micro-chips I am sure you have received some of those. To what extent do these messages deter you from taking the vaccine?

Do you think that asylum-seekers and migrants are also being considered in the COVID-19 vaccine? Why do you say so?

What do you think the South African government could do to encourage foreigners to take the vaccine?

What are some of the things that you think should be done to encourage foreigners to take the COVID-19 vaccine?

Are there other things that make you not to take or not willing to take the COVID-19 vaccine?

Is there a relationship between your documentation status and taking the COVID-19 vaccine?

Are there any other reasons why you did not take the vaccine that you will like to tell us?
